# Extraction, Encapsulation into Lipid Vesicular Systems, and Biological Activity of *Rosa canina* L. Bioactive Compounds for Dermocosmetic Use

**DOI:** 10.3390/molecules27093025

**Published:** 2022-05-08

**Authors:** Valentina Sallustio, Ilaria Chiocchio, Manuela Mandrone, Marco Cirrincione, Michele Protti, Giovanna Farruggia, Angela Abruzzo, Barbara Luppi, Federica Bigucci, Laura Mercolini, Ferruccio Poli, Teresa Cerchiara

**Affiliations:** 1 Drug Delivery Research Lab., Department of Pharmacy and Biotechnology, Alma Mater Studiorum, University of Bologna, Via San Donato 19/2, 40127 Bologna, Italy; valentina.sallustio2@unibo.it (V.S.); angela.abruzzo2@unibo.it (A.A.); barbara.luppi@unibo.it (B.L.); federica.bigucci@unibo.it (F.B.); 2 Pharmaceutical Botany Lab., Department of Pharmacy and Biotechnology, Alma Mater Studiorum, University of Bologna, Via Irnerio 42, 40127 Bologna, Italy; ilaria.chiocchio2@unibo.it (I.C.); manuela.mandrone2@unibo.it (M.M.); ferruccio.poli@unibo.it (F.P.); 3 Pharmaco-Toxicological Analysis (PTA Lab.), Department of Pharmacy and Biotechnology, Alma Mater Studiorum, University of Bologna, Via Belmeloro 6, 40126 Bologna, Italy; marco.cirrincione2@unibo.it (M.C.); michele.protti2@unibo.it (M.P.); laura.mercolini@unibo.it (L.M.); 4 Pharmaceutical Biochemistry Lab., Department of Pharmacy and Biotechnology, Alma Mater Studiorum, University of Bologna, Via San Donato 19/2, 40127 Bologna, Italy; giovanna.farruggia@unibo.it

**Keywords:** *Rosa canina* L. extract, polyphenols, liposomes, hyalurosomes, ethosomes, cosmetic ingredients, antioxidant activity, skin retention, cytotoxicity

## Abstract

Valorization of wild plants to obtain botanical ingredients could be a strategy for sustainable production of cosmetics. This study aimed to select the rosehip extract containing the greatest amounts of bioactive compounds and to encapsulate it in vesicular systems capable of protecting their own antioxidant activity. Chemical analysis of *Rosa canina* L. extracts was performed by LC-DAD-MS/MS and ^1^H-NMR and vitamins, phenolic compounds, sugars, and organic acids were detected as the main compounds of the extracts. Liposomes, prepared by the film hydration method, together with hyalurosomes and ethosomes, obtained by the ethanol injection method, were characterized in terms of vesicle size, polydispersity index, entrapment efficiency, zeta potential, in vitro release and biocompatibility on WS1 fibroblasts. Among all types of vesicular systems, ethosomes proved to be the most promising nanocarriers showing nanometric size (196 ± 1 nm), narrow polydispersity (0.20 ± 0.02), good entrapment efficiency (92.30 ± 0.02%), and negative zeta potential (−37.36 ± 0.55 mV). Moreover, ethosomes showed good stability over time, a slow release of polyphenols compared with free extract, and they were not cytotoxic. In conclusion, ethosomes could be innovative carriers for the encapsulation of rosehip extract.

## 1. Introduction

Currently, the valorization of wild plants defined as “plants that are gathered (not cultivated), even if some of them may grow on cultivated rather than on uncultivated or forest land” [1], could represent a strategy to obtain sustainable products preserving biodiversity. According to the 2030 Agenda for Sustainable Development, item 12 “ensure sustainable production and consume” and item 15 “protect, restore, and promote sustainable use of terrestrial ecosystem” could be also approached by the careful use of natural resources in the cosmetic field. 

*Rosa canina* L. is a wild plant from the *Rosaceae* family native to Europe and Western Asia [2]. As it is a spontaneous shrub, soil processes or fertilizations are not required for its growth. However, it was also planted to delimitate cultivated areas or for ornamental purposes, or as rootstock for other cultivated species [3]. *Rosa canina* L. pseudofruits, known as rosehips, have been used since ancient times to produce syrups, jams, and liqueurs, but they are also well-known medicinal berries used in folk remedies. Vitamins and polyphenols contained in rosehips alleviate various dermal disorders, including inflammation, infections, and skin irritations [4].

Among all hydrosoluble phytochemicals contained in rosehips, the highest antioxidant activity can be attributed to ascorbic acid and polyphenols [5]. In fact, it has been demonstrated that rosehips have the highest content of ascorbic acid among fruits and vegetables [4]. This vitamin is widely used in antiaging cosmetics for its antioxidant and antinflammatory properties and its contribution to collagen synthesis.

Phenolic compounds are secondary metabolites synthesized by plants to improve their protection against pathogens and ultraviolet radiation. Various authors reported that rosehips are rich in polyphenols in particular catechin, gallic acid, quercetin, and cyanidin [5]. Unfortunately, some of these useful bioactive compounds are not stable for long periods, and these beneficial properties could be lost [6]. 

Encapsulation in nanocarriers can preserve the biological activity of useful compounds which prevent oxidation and photolysis and can improve the bioavailability of polyphenolic compounds characterized by poor water solubility. Moreover, it is known that lipid vesicles improve penetration of bioactive compounds through the different layers of the skin, and, for this reason, liposomes are used for skin delivery of cosmetics. Liposomes are lipid vesicles with variable sizes, ranging from 20 nm to several micrometers, characterized by a double layer of phospholipids that permit the inclusion of polar substances in the aqueous cavity and nonpolar substances between the bilayer [7,8,9]. Moreover, liposomes offer several advantages, including biocompatibility, capacity for self-assembly, and a wide range of physicochemical and biophysical properties that can be modified to control their biological activities [10]. Besides conventional liposomes, novel carriers including hyalurosomes and ethosomes have been investigated for the dermocosmetic delivery of plant ingredients [11]. Hyalurosomes are lipid vesicles immobilized by sodium hyaluronate and have been developed to promote skin deposition and therapeutic efficacy of low bioavailable compounds, such as curcumin [12] and the phenolic extracts from licorice (*Glycyrrhiza glabra* L.) roots [13]. Ethosomes were formulated by Touitou [14] and are soft and flexible lipid vesicles made of phospholipids, water, and from 20 to 50% ethanol. Ethanol can stabilize the lipid vesicles as it ameliorates the solubilization of the extracts and can enhance permeation and retention of the bioactives in the different layers of the skin [15]. Compared to conventional liposomes, they offer better features, such as ultraflexible structure, higher stability, and smaller vesicle sizes for the efficient delivery of bioactive compounds to the skin [16]. Thanks to these characteristics, some ethosomal formulations based on anticellulite and antiaging agents are currently marketed in the cosmetics field. 

The first aims of this work were to extract and evaluate the chemical composition (ascorbic acid and phenolic compounds) and the antioxidant activity of pseudofruits of *Rosa canina* L. growing naturally near Brisighella (Italy) and to preserve the bioactive compounds using different methods such as freezing, air-drying, and lyophilization. Secondly, rosehip extract was encapsulated in nanovesicles to avoid oxidation and to preserve biological activities for cosmetic use. Specifically, rosehip extract-loaded liposomes, hyalurosomes, and ethosomes were prepared and characterized for their physicochemical properties, such as vesicle size (VS), polydispersity index (PDI), entrapment efficiency (EE), zeta potential (ζ), in vitro release, and skin retention. In addition, a biocompatibility assay was performed on WS1 fibroblasts.

## 2. Results and Discussion

### 2.1. Extraction and Analysis of Bioactive Compounds from Rosehip 

#### 2.1.1. Preparation of Rosehip Extract

To select an extract rich in bioactive compounds, rosehips were preserved by different methods including freezing, drying, and lyophilization. According to Um et al. [17], a solution of H_2_O:EtOH 50:50 *v*/*v* and ultrasound-assisted extraction were used to extract the highest amount of antioxidant compounds. In particular, ethanol has the ability to extract phenolics and flavonoids, while water facilitates the extraction of phenolic acids and promotes the swelling of the vegetal matrix [18]. For this reason, the use of an ethanol-water mixture is more effective than water in extracting a high amount of antioxidant compounds [19]. After the concentration of hydroalcoholic solutions, three different rosehip extracts were obtained, namely frozen rosehip (RCF), dried rosehip (RCD) and lyophilized rosehip (RCL). The yield of extraction was 0.35 ± 0.02 g extract/g for RCF, 0.39 ± 0.06 g extract/g for RCD, and 0.48 ± 0.05 g extract/g for RCL.

#### 2.1.2. LC-DAD-MS/MS Analysis

Table 1 shows the content of ascorbic acid and phenolic compounds obtained from rosehips preserved by different methods. All principal biochemical compounds were detectable in the prepared extracts and displayed some difference in their concentrations.

Generally, the content of all bioactive compounds extracted from rosehips followed the order: RCL > RCD > RCF. This is probably due to the more efficient removal of water from the raw materials for lyophilized rosehips that guarantees a more dehydrated final extract. Similar results for preserved rosehips were found by Tabaszewska et al. [20].

Ascorbic acid is a reference substance for antioxidant activity. Among all prepared extracts, RCL extract showed the highest content of ascorbic acid (5.59 ± 0.36 µg/mg), confirming that lyophilization could be considered an effective preservation method to recover a high amount of this vitamin.

Phenolic substances are well known for their antioxidant properties [21]. All the investigated polyphenols demonstrated additional properties to the antioxidant activity. In particular, catechins are photoprotective and antibacterial, cyanidin has antinflammatory and depigmentation properties, quercetin inhibits tyrosinase [22], and gallic acid is helpful for atopic dermatitis and hyperpigmentation thanks to its antiinflammatory and antimicrobial properties [23]. Among the phenolic compounds of analyzed extracts and according to Olsson et al. [24] and Liaundaskas et al. [21], catechin was identified as the major phenolic in RCL extract (12.27 ± 0.74 µg/mg). Another abundant compound identified in RCL extract was cyanidin (6.87 ± 0.42 µg/mg), and according to the literature, this is the most common compound among all anthocyanins [25]. Moreover, the RCL extract showed also a high amount of quercetin (4.38 ± 0.30 µg/mg), which is a potent antioxidant flavonol even if its poor water solubility can limit its bioavailability [22]. Finally, the amount of gallic acid, which is the reference compound for the determination of polyphenols, was 1.12 ± 0.13 µg/mg in RCL extract.

This chemical composition of rosehip extracts depended not only on the preservative method and extraction process, but also on the genetic factors of plants, the pedoclimatic condition of growing, the degree of ripeness, and time of harvesting. Many previous studies noticed this quantitative and qualitative variability in the composition of rosehips [26]. In particular, *R. canina* plants suffering from drought conditions or high temperatures seem to increase the production of these bioactive compounds as a defense mechanism [27]. 

To conclude, even if all antioxidants were detectable in all three types of extracts, the one obtained from lyophilized rosehip displayed a large amount of all investigated substances. Taking into account these considerations and according to Tabaszeskwa [20] and Fascella [25], rosehip lyophilization can be considered the most adequate preservative process, as it guarantees a higher content of bioactive substances.

#### 2.1.3. Total Phenolic Content, Total Flavonoid Content, and Antioxidant Activity 

Total Phenolic Content (TPC), Total Flavonoid Content (TFC), and antioxidant activity (AA%) are shown in Table 2. The results of TPC were expressed as μg of gallic acid equivalent (GAE) per mg of extract, and the results of TFC were reported as μg of quercetin equivalent (μg QE) for mg of extract.

The TPC was determined spectrophotometrically using the Folin–Ciocalteu method. According to the results of LC-DAD-MS/MS analysis, among all the extracts, the RCL extract showed the highest content of TPC (128.63 ± 1.03 μg GAE/mg extract; *p* < 0.05), agreeing with the data reported by Murathan et al. [28] and Fascella et al. [25]. 

The TFC was determined by a UV–vis spectrophotometric assay, based on the ability of the aluminum ion to form a complex with carbonyl and hydroxyl groups of flavonoids [29]. According to previous data, RCL possessed a large amount of flavonoids, evaluated as a TFC value of 26.43 ± 0.18 μgQE/mg extract. Tabaszewska et al. [20] and Um et al. [17] reported a similar trend in the value of TPC and TFC for frozen, hot-dried, and lyophilized rosehips, confirming that lyophilization represents a valuable preservation method for bioactive compounds. 

Finally, the AA of rosehip extracts was determined using DPPH assay. As reported in Table 2, among all the extracts, RCL extract showed the highest antioxidant activity (88.83 ± 0.70%). Generally, the antioxidant activity could be attributed primarily to the presence of phenolic substances and ascorbic acid in the extract. It can be noticed that although RCD extract contained a larger amount of polyphenols and flavonoids than RCF extract, the AA of RCD extract was lower than those of RCF extract and it is probably due to the oxidation of ascorbic acid during the long process of air drying. Similar findings were reported by Rutwoskwa et al. [30] and Tabaszewska et al. [20].

In summary, RCL extract showed the largest amount of bioactive compounds and the corresponding highest antioxidant activity; for this reason, we selected it for the following analysis and for encapsulation in lipid vesicles.

#### 2.1.4. ^1^H-NMR Analysis

**Figure 1:**
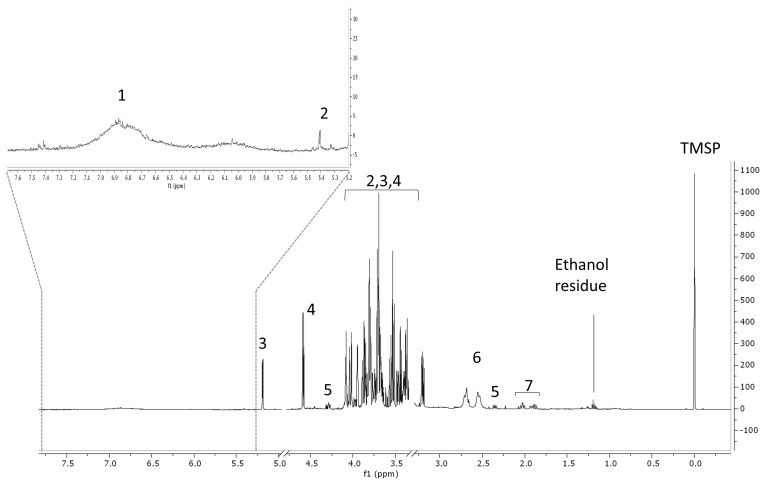
^1^H−NMR profiling of RCL extract. 1 = aromatic compounds, 2 = sucrose, 3 = α−glucose, 4 = β−glucose, 5 = malic acid, 6 = citric acid, 7 = quinic acid. TMSP = tetramethylsylilpropionic acid (internal standard).

An overview of the most abundant metabolites in the RCL extract was obtained by ^1^H-NMR profiling. This technique proved useful to quickly detect primary and secondary metabolites in complex matrices, such as plant extracts, and was successfully employed in metabolomics for different purposes, i.e., quality control [31] or to compare different drying methods [32]. According to this analysis, the extract was rich in glucose; in fact, both α- and β-glucose anomeric protons, whose diagnostic signals were a doublet at δ 5.19 (*J* = 3.77 Hz) and a doublet at δ 4.59 (*J* = 7.88 Hz), respectively (Figure 1), were evident. Organic acids were also detected, namely malic acid, citric acid, and quinic acid. Between δ 6.6 and 7.1, an overlapping of signals was observed, which could be due to tannins, especially gallotannins, whose repetition of gallic acid moieties generated several singlets around δ 7 [33]. Tannins are phytochemical compounds already found in *Rosa canina* L. [34].

#### 2.1.5. FT-IR Analysis

**Figure 2:**
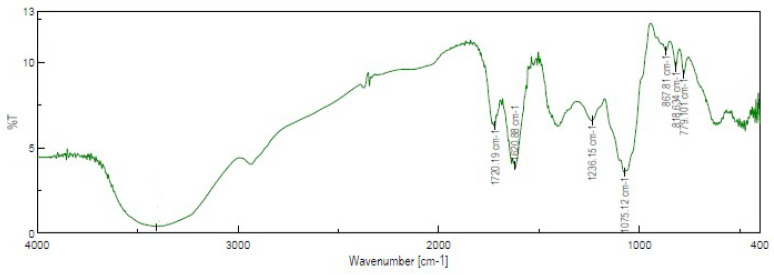
FT−IR spectrum of RCL extract.

The functional groups of bioactive compounds contained in RCL extract such as flavonoids, polyphenols, sugars, and acids, were investigated by FT-IR spectroscopy and their absorbances at specific wavelengths are shown in Figure 2. FT-IR spectrum of RCL extract revealed the typical band of polysaccharides in the region of 1200–1000 cm^−1^, which is attributed to the stretching vibration of the side groups (C-OH) and the vibrations of the glycosidic bond (C-O-C) in the polysaccharide chains [35]. The band identified at 3400 cm^−1^ indicates a specific stretching vibration of the O-H groups of polyflavonoids. The absorption bands for the carboxyl group and the ester group of the extract can be found at 1720–1745 cm^−1^ and are related to the presence of organic acids [36], while the band at 1750–1550 cm^−1^ corresponds to the carbonyl group C=O of fructose and aldehyde group CH=O of glucose. Another interesting region is represented by bands in the range from 890 to 760 cm^−1^, which corresponds to the specific oscillation of the anomeric region of carbohydrates. Peak absorption at 518 cm^−1^ shows the presence of anthocyanin pigments.

### 2.2. Encapsulation of Lyophilized Rosehip Extract in Lipid Vesicles

RCL extract was encapsulated into lipid vesicles to protect it from oxidation and to improve the skin absorption of bioactive substances. Lipid vesicles are among the most attractive drug carriers thanks to their structural similarity to skin barriers and compatibility with the majority of bioactive compounds [6]. In particular, the extract was encapsulated in liposomes (LP) and hyalurosomes (HYA), by using the film hydration method, and in ethosomes (ET) by using the ethanol injection method.

### 2.3. Vesicles Characterization

The physicochemical properties of nanocarriers can influence the in vitro and in vivo behavior and consequently their cosmetic application [10]. The prepared lipid vesicles were characterized in terms of vesicles size (VS), polydispersity index (PDI), ζ potential (mV), and entrapment efficiency (EE), as reported in Table 3.

The sizes of unloaded LP and HYA were 375 ± 27 nm and 451 ± 19 nm, respectively. According to the literature, the higher size of HYA compared to LP (*p* < 0.05) could be attributed to the absorption of sodium hyaluronate onto the surface of lipid vesicles and its intercalation between the phospholipid bilayer [22]. Regarding ET obtained by the ethanol injection method, unloaded vesicles showed VS equal to 127 ± 1 nm. Among all the vesicles studied, ET presented the lowest size (*p* < 0.05). This result could be attributed to the composition of ET and it is in line with previous studies indicating that a high percentage of ethanol (30%) and a low percentage of PC (1%) lead to the formation of vesicles with minimum size [11]. 

Concerning loaded vesicles, LP and HYA were characterized by size values equal to 1971 ± 191 nm and 2236 ± 68 nm, respectively. According to the literature, LP and HYA loaded with RCL extract could be considered “giant liposomes”, as their dimensions were between 400 and 2500 nm [37]. This enlargement could be attributed to the incorporation of phytochemical compounds in vesicles structures. In particular, as demonstrated by the analysis described in Section 2.1, RCL extract contained sugars, organic acids, polyphenols, and vitamins having different polarities. Thus, the nonpolar compounds were distributed in the bilayer and the polar ones in the aqueous and polar parts of vesicles, and this distribution could provide an increase in size [6]. In the case of ethosomes, the size increase between unloaded and loaded vesicles was less evident (from 127 ± 1 nm to 196 ± 2 nm) owing to the presence of ethanol, which improved the extract solubilization and facilitated its encapsulation in vesicles [11]. 

Regarding PDI measurements, ethosomal formulations showed a lower PDI value than liposomes and hyalurosomes (*p* < 0.05), indicating that the presence of ethanol in the formulation could also refine the homogeneity of the vesicular system [38].

The value of zeta potential expresses the degree of repulsion between the vesicles; thus, it is predictive of the stability of the vesicular systems. For all prepared formulations, this parameter had a negative value owing to the presence of the phosphate functional group of phosphatidylcholine. In particular, according to the literature, hyalurosomes’ ζ potential was more negative than that of liposomes and ethosomes (*p* < 0.05) due to the presence of sodium hyaluronate on the surface of vesicles [13]. The value of ζ potential for unloaded ethosomes was less negative than of LP and HYA (*p* < 0.05), probably due to the presence of ethanol, in agreement with other findings. In particular, Andleeb et al. [39] reported that ζ potential increased with the increase of a percentage of ethanol.

A similar trend was observed for the loading vesicles. In particular, the presence of the extract in the vesicles led to an increase in ζ potential. Among other factors, it could be attributed to organic acids of extract that could interact with phosphatidylcholine neutralizing in part the negative charge of this zwitterionic molecule.

Entrapment efficiency is a predictive parameter to evaluate the potentiality of a delivery system. LP and HYA showed an EE% of 65.50 ± 0.29 and 63.97 ± 0.35, respectively, while a higher EE% (92.30 ± 0.02) was observed for ET (*p* < 0.05). This behavior was probably due to the effect of ethanol, as previously described. The ability of ET to load high amounts of natural extract was also referred for *Sambucus nigra* extract by Pavaiolu et al. [6], grape extract by Manca et al. [18], and licorice extract by Castangia et al. [13]. 

Based on this data, among all the prepared formulations, ET presented an optimal nanometric dimension that can improve skin penetration, narrow size distribution and the highest EE of RCL extract. For these reasons, this formulation was selected for the following studies.

### 2.4. Vesicles Physical Stability

**Figure 3:**
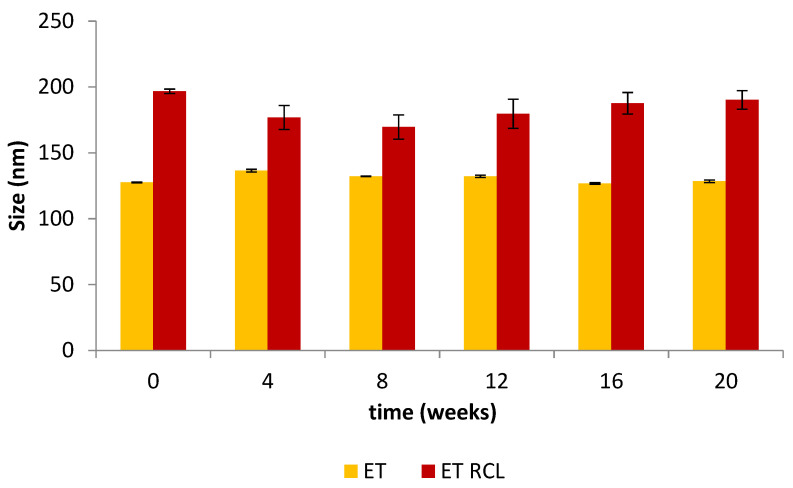
Size of unloaded (ET) and loaded ethosomes (ET RCL) during 20 weeks of storage at 4.0 ± 1.0 °C.

The stability of nanocarriers is fundamental in guaranteeing the effectiveness and the safety of a cosmetic formulation [38]. Figure 3 shows the variation in the size of unloaded ET and loaded ET (ET RCL) over a storage period of 20 weeks at +4.0 ± 1.0 °C. No relevant changes in the size of ET were detected over this period; it remained less than 300 nm, which is favorable to skin penetration [14].

### 2.5. In Vitro Release Studies 

The release profile of the bioactive compounds for skincare cosmetics can be useful in understanding their absorption in the skin layers before rubbing or washing [40]. The release of polyphenols from ET RCL and the free extract solution (1 mg/mL, RCL solution) was performed in a mixture of PBS:EtOH 7:3 *v*/*v* at a temperature of 32 ± 1 °C. The Folin–Ciocalteu test was used to determine the TPC released over a period of 6 h.

**Figure 4:**
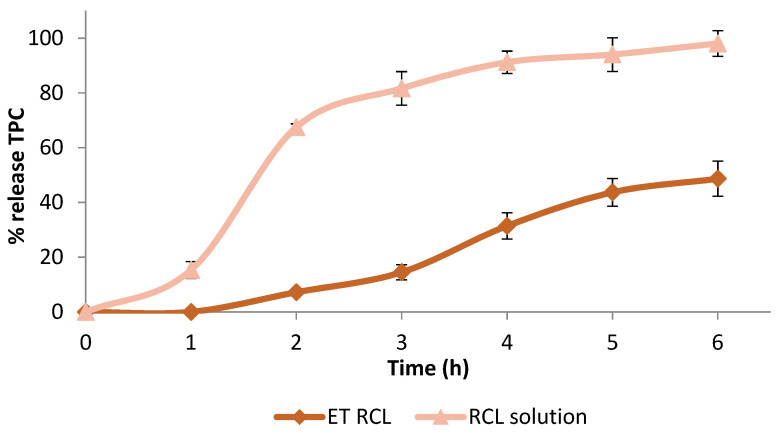
In vitro release of TPC in PBS:EtOH (7:3 *v*/*v*) from ET RCL and RCL solution.

In Figure 4, the experimental data for in vitro release test are presented as a percentage amount of TPC released over time. The free extract exhibited a fast release with 67.48 ± 1.20% of TPC after 120 min and reached the maximum released percentage amount (98.07 ± 4.69%) after 6 h. On the other hand, the extract encapsulated in lipid vesicles showed a slow release over the time, and after 6 h, the amount of TPC released was 48.65 ± 6.42%. This finding is in accordance with similar studies reported by Pavaloiu et al. [6]. 

To evaluate release mechanisms and kinetics, the in vitro release data were fitted to different kinetics models: zero-order, first-order, Higuchi, and Korsmeyer–Peppas. Table 4 shows the parameters of fitted release data. The results revealed that the release of TPC from ethosomes follows zero-order release, providing a constant release of TPC over time.

### 2.6. In Vitro Skin Retention Studies from Ethosomes 

**Figure 5:**
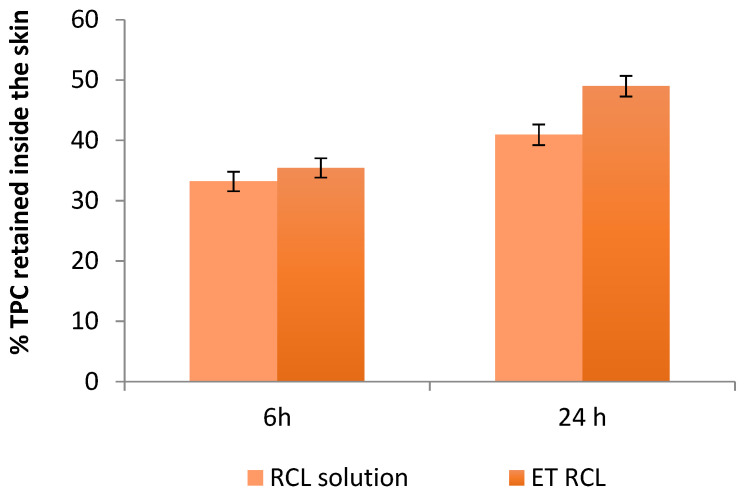
In vitro skin retention of TPC from RCL solution and ET RCL after 6h and 24 h.

The ability of a delivery system to improve bioactive substance absorption and retention inside skin layers could be assessed by in vitro skin retention studies [40]. Figure 5 compares the percentage of TPC from loaded ethosomes or free extract detectable in porcine skin after 6 h and 24 h of permeation. 

The TPC percentages of ET RCL retained inside the skin after 6 h and 24 h were 35.42 ± 0.16% and 48.98 ± 1.77%, respectively. For the free extract, used as a control, the TPC percentages retained inside the skin after 6 h and 24 h were 33.18 ± 0.17% and 40.92 ± 1.34%, respectively. These results suggested that ET RCL favored the retention of TPC in the skin after 24 h with respect to the control (*p* < 0.05). In fact, ethanol is supposed to enhance skin permeation by increasing the fluidity and permeability of the lipids of the *stratum corneum* bilayer [11]. For this reason, ethanol improved the performance of vesicles as carriers for the delivery of bioactive compounds to the skin [41]. 

Moreover, the skin permeation experiments showed that polyphenols did not reach the receptor compartment after 24 h, suggesting that the formulation is suitable for dermo-cosmetic use. Zillig et al. [42] indicated that polyphenols concentrated in the epidermis and dermis are favorable for the antiaging action of skincare. 

Moreover, the antioxidant activity by the DPPH assay on the suspension recovered from the donor compartment after the permeation test was performed. The AA% determined in the suspension after 6 h was 80.70 ± 2.61% for the encapsulated extract and 15.50 ± 2.18% for the free extract, suggesting additionally that free extract is more exposed to oxidation during the application, losing part of its antioxidant activity. 

### 2.7. In Vitro Cytotoxicity Assay 

The biocompatibility of ET was tested on WS1 fibroblasts which are some of the most representative cells of skin tissue and the target of the topical formulation [18]. The MTT assay is based on the reduction of the tetrazolium salt into formazan by cellular dehydrogenases—in particular, the mitochondrial ones. The absorption at 570 nm of the formed formazan is proportional to the number of viable, metabolically active cells. For this reason, this assay is considered an estimate of cell viability, proliferation, and cytotoxicity.

**Figure 6:**
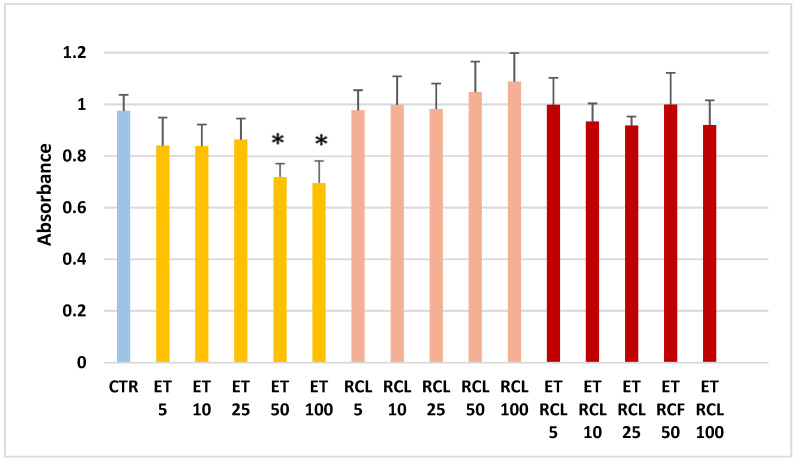
MTT assays on WS1 cells treated for 24 h with increasing concentration of ET, RCL solution, and ET RCL. Effects on cell viability were tested in comparison with control cells (* *p* < 0.05).

The WS1 fibroblasts were treated for 24 h with the RCL free extract and ET and unloaded and loaded with the RCL extract in a range of concentrations between 5 and 100 µg/mL. The MTT assay, reported in Figure 6, evidences that WS1 viability is not affected by the RCL extract free or loaded in the ethosomes. Instead, the unloaded ET showed a slight detrimental effect which was statistically significant at the two highest concentrations, 50 and 100 µg/mL. The toxic effects of the unloaded vehicles at the highest concentrations is well-known [43,44,45].

Although the RCL extract seemed to stimulate cell vitality more than the encapsulated extract, we must consider that the free extract quickly lost its antioxidant power, which was instead preserved in the ethosomes. Therefore, we can consider that the proposed formulation is advantageous compared to the free extract. 

## 3. Materials and Methods

### 3.1. Materials

Rosehips were collected in November 2020 from wild plants around Brisighella (RA, Emilia Romagna, Italy), GPS coordinates 44°12′46.9″ N 11°50′46.6″ E. L-α-phosphatidylcholine from egg yolk and all the solvents were purchased from Sigma-Aldrich (Milan, Italy). Sodium hyaluronate (molecular weight = 800–1200 kDa) was sourced from Farmalabor (Canosa di Puglia, Italy). Folin–Ciocalteu reagent was sourced from Titolchimica (Pontecchio Polesine, Italy). All other chemicals were purchased from Carlo Erba (Milan, Italy). Deuterium oxide (D2O, 99.90% D) and CD3OD (99.80% D) were purchased from Eurisotop (Cambridge Isotope Laboratories, Inc, Saclay, France). Standard 3-(trimethylsilyl)-propionic-2,2,3,3-d4 acid sodium salt (TMSP), sodium phosphate dibasic anhydrous, and sodium phosphate monobasic anhydrous and Dulbecco’s modified Eagle medium supplemented with D-glucose were purchased from Sigma-Aldrich Co. (St. Louis, MO, USA). Fetal bovine serum (FBS), L-glutamine, penicillin (1000 U/mL) and streptomycin were purchased from Euroclone S.p.A., Milan, Italy). Phosphate buffer solution at pH 7.4 (PBS) was prepared with the following composition: 2.98 g/L Na_2_HPO_4_ × 12 H_2_O, 0.19 g/L KH_2_PO_4_, 8 g/L NaCl. 

### 3.2. Preparation of Rosehip Extracts

Rosehips were treated using different methods of preservation such as freezing, air-drying, and lyophilization. Freezing was performed in a commercial freezer at −20 °C, air-drying was performed in the dark at room temperature for 20 days, and lyophilization was performed with a vacuum freeze-dryer (Christ Freeze Dryer ALPHA 1-2, Milan, Italy) at 0.01 atm and −48 °C for 72 h.

The seeds were removed from differently preserved rosehips and their pulp was finely chopped; 10 g of pulp was put in a mixture of ethanol/water 50:50 *v*/*v* (150 mL) and sonicated (Transonic TP690 by Elma, Singen, Germany) for 40 min at room temperature. After extraction, the mixture was centrifuged at 4000 rpm for 20 min and the supernatants were filtered using a Buchner filter (Rotofix 32A by Hettich, Tuttlingen, Germany). The residue was further extracted by repeating the procedure mentioned above. The supernatants were collected and evaporated under a vacuum at 40 °C (Buchi Rotavapor Heating Bath B-490) to remove alcohol and most of the water. The concentrated extract was freeze-dried at 48 °C for 48 h and stored at +4.0 ± 1.0 °C in the dark until use. 

### 3.3. Rosehip Extracts Characterization

#### 3.3.1. LC-DAD-MS/MS Analysis 

Rosehip extracts were analyzed using an original LC-DAD-MS/MS method developed and fully validated ad hoc for the purpose of this study. Analyses were carried out using a Waters (Milford, MA, USA) Alliance e2695 LC system coupled to a Waters 2998 diode array detector and a Waters Micromass Quattro Micro triple quadrupole mass spectrometer. Separations were obtained on a Restek (Bellefonte, PA, USA) Ultra AQ RP C18 column (50 mm × 2.1 mm; 3 μm). Mass spectrometry acquisition was carried out in multiple reaction monitoring (MRM) using an electrospray ionization (ESI) source with negative polarity (ESI-). Due to the different physicochemical properties of the analytes, two different analytical conditions were used for the separation of phenolic compounds (cyanidin, gallic acid, (+)-catechin and quercetin) and for the quantitation of ascorbic acid. 

For the analysis of phenolic compounds, the mobile phase was composed of 0.1% (*v*/*v*) FA in acetonitrile (Solvent A) and 0.1% (*v*/*v*) FA in ultrapure water (solvent B). The gradient program started with 2% of solvent A, maintained constant for 3 min then raised linearly to 15% in 2 min. This condition was kept constant for 4 min before decreasing linearly to the starting condition in 5 min. Then the column was re-equilibrated with 2% of solvent A for 2 min. The flow rate was set at 0.4 mL/min. The following MS/MS parameters were used for the identification and quantitation of phenolic compounds: ion source voltage, 3 kV; ion source temperature, 130 °C; desolvation temperature, 350 °C; desolvation gas flow, 600 L/h; scan duration: 0.3 s; desolvation gas: nitrogen; collision gas: argon. MRM transitions exploited for the quantitation of the target analytes were: cyanidin, [M-H-] 287.11 → 121.2 (cone voltage: 20 V, collision energy: 15 V); gallic acid, [M-H-] 168.89 → 124.9 (cone voltage: 26 V, collision energy: 10 V); (+)-catechin, [M-H-] 291.01 → 138.9 (cone voltage: 20 V, collision energy: 14 V); quercetin, [M-H-] 301.02 → 150.9 (cone voltage: 27 V, collision energy: 22 V). DAD acquisition was carried out in the 200–400 nm wavelength range while monitoring different wavelengths for each compound (285 nm for cyanidin; 270 nm for gallic acid; 260 nm for (+)-catechin; 370 nm for quercetin).

The analysis of ascorbic acid was carried out by means of an isocratic mobile phase composed of 0.1% (*v/v*) FA in methanol and 0.1% (*v/v*) FA in ultrapure water (15/85, *v/v*) at a flow rate of 0.3 mL/min while exploiting the same chromatographic column used for the analysis of phenolic compounds. MS/MS parameters for ascorbic acid analysis were the following: ion source voltage, 4 kV; ion source temperature, 115 °C; desolvation temperature, 300 °C; desolvation gas flow, 300 L/h; scan duration: 0.3 s; desolvation gas: nitrogen; collision gas: argon. The MRM transition used for ascorbic acid analysis was [M-H-] 175.13 → 115.2 (cone voltage: 20 V; collision energy: 15 V), while the monitored wavelength was 251 nm. The injection volume was 10 μL for both of the two analytical systems.

#### 3.3.2. Total Phenolic Content (TPC) 

The TPC was determined by using the Folin–Ciocalteu reagent according to Singleton et al. [46]. Briefly, 0.2 mL of a water solution of extract (1 mg/mL) was added to 1.00 mL of 1:10 diluted Folin–Ciocalteu’s phenol reagent, followed by the addition of 0.8 mL of sodium carbonate solution (7.5% *w*/*v*). After 30 min in the dark at +40.0 ± 1.0 °C, the absorbance at 750 nm was measured spectrophotometrically (UV-Vis 1601 spectrophotometer, Shimadzu, Milan, Italy). Distilled water was used as a blank. TPC was estimated from a standard curve of gallic acid (R^2^ = 0.999). All measurements were performed in triplicate and results were expressed as gallic acid equivalent in μg/mg rosehip extract (μg GAE/mg extract). 

#### 3.3.3. Total Flavonoid Content (TFC)

The TFC was determined using the pharmacopeial method with minor modifications [47]. Briefly, 0.1 mL of 5% (*w*/*v*) AlCl_3_ solution was added to 0.9 mL of the extract solution (1 mg/mL). After incubation at room temperature for 30 min in the dark, the absorbance was measured at 430 nm. The TFC was estimated from a standard curve of quercetin (R^2^ = 0.999) and was expressed as μg of quercetin equivalent (μg QE)/mg of extract. Measurements were performed in triplicate.

#### 3.3.4. Antioxidant Activity (AA) 

The AA was determined by the 2,2′-di-phenyl-1-picrylhydrazyl radical (DPPH, Sigma Aldrich) reduction assay as described by Brand-Williams et al. [48] with minor modification. Briefly, different concentrations of each extract as well as ascorbic acid (used as standard antioxidant compound) were mixed with a solution of DPPH (0.1 mM in methanol) at room temperature. The mixtures were kept in the dark for 30 min and the absorbance was measured at 517 nm. Methanol was used as a blank solution and DPPH solution was the control. The test was carried out in triplicate. Results are expressed as percentage of inhibition of the DPPH radical according to the following equation: Inhibition % = [(A_0_ − A)/A_0_] where A_0_ was the absorbance of DPPH control and A was the absorbance of the sample with DPPH.

#### 3.3.5. ^1^H-NMR Analysis 

The RCL extract was solubilized at the concentration of 10 mg/mL in a blend of solvents constituted by phosphate buffer (90 mM; pH 6.0) in H_2_O-d_2_ (containing 0.01% TMSP) and MeOH-d_4_ (1:1) and subjected to ^1^H-NMR analysis. The spectrum was recorded at 25 °C on a Varian Inova instrument (equipped with a reverse triple resonance probe) (Agilent, Santa Clara (CA), United States) operating at a ^1^H-NMR frequency of 600.13 MHz, and MeOH-d_4_ was used as an internal lock. The spectrum consisted of 256 scans (corresponding to 16 min) with a relaxation delay (RD) of 2 s, acquisition time of 0.707 s, and spectral width of 9595.8 Hz (corresponding to δ 16.0). A presaturation sequence (PRESAT) was used to suppress the residual water signal at δ 4.83 (power = −6 dB, presaturation delay 2 s). The spectra were manually phased and baseline corrected and were calibrated to the internal standard trimethyl silyl propionic acid sodium salt (TMSP) at δ 0.0 using Mestrenova software (Mestrelab Research, Santiago de Compostela, Spain).

#### 3.3.6. FT-IR Analysis 

FT-IR spectra were performed with a Jasco FT-IR-4100 spectrophotometer (Jasco, Lecco, Italy) using the KBr pellet technique. The sample was prepared in agave mortar by mixing the RCL extracts with KBr (1:9 *w*/*w*). Measurements were carried out on the infrared scale of 400–4000 cm^−1^.

### 3.4. Encapsulation of Rosehip Extract in lipid vesicles

#### 3.4.1. Preparation of Liposomes and Hyalurosomes 

Liposomes and hyalurosomes were prepared using the thin layer film evaporation method reported by Uchino et al. [49] with some modification. Firstly, 300 mg of L-α-phosphatidylcholine was dissolved in 10 mL of an organic mixture of CHCl_3_-CH_3_OH 9:1 in a 500 mL round-bottomed flask and stirred at 120 rpm for 10 min. After dissolution, the organic mixture was evaporated using the rotatory evaporator (Buchi Rotavapor R-200, Flawil, Switzerland) at 55 °C under a reduced pressure of −0.8 atm for 30 min with a constant stirring at 210 rpm. The dried lipid film was subsequently hydrated with a solution of 40 mL of PBS buffer (pH 7.4) containing 40 mg of rosehip extract to obtain loaded liposomes. Hyalurosomes were obtained through rehydration of the lipid film with a sodium hyaluronate solution prepared by dissolving the polymer (20 mg) in 40 mL of PBS for 30 min and containing 40 mg of rosehip extract. The mixture was stirred for 60 min at 210 rpm at room temperature using small glass spheres to ease the formation of the vesicles. The resulting suspension was extruded 15 times through a 200 µm pore-sized polycarbonate membrane (LiposoFast manual syringe extruder, Avestin Europe GmbH, Mannheim, Germany). Unloaded vesicles were prepared as a control.

#### 3.4.2. Preparation of Ethosomes

Ethosomes were prepared by the ethanol injection-sonication method reported by Ma et al. [50] with minor modifications. Briefly, L-α-phosphatidylcholine (100 mg) was dissolved in ethanol (3 mL) in a covered beaker to avoid ethanol evaporation. The rosehip extract (10 mg) was dissolved in double-distilled water (7 mL) and mixed uniformly with a magnetic stirrer. The ethanolic solution of phospholipid was slowly (1 mL/min) added to the aqueous solution with a syringe under constant stirring at 700 rpm. The resulting vesicle suspension was homogenized with an ultrasonic probe (Transonic T310, Elma, Singen, Germany) for 15 min. Unloaded ethosomes were prepared as control. 

### 3.5. Vesicles Characterization

#### 3.5.1. Size and Zeta Potential Measurements 

The prepared lipid vesicles were characterized for their vesicle size (VS) and their polydispersity index (PDI). The vesicle suspensions were diluted (1:800 *v*/*v*) in ultrapure water (18.2 M Ώ cm, MilliQ apparatus by Millipore, Milford, MA, USA). Size and PDI were measured by PCS (photon correlation spectroscopy) using a Brookhaven 90-PLUS instrument (Brookhaven Instruments Corp., Holtsville, NY, USA) with a He-Ne laser beam at a wavelength of 532 nm (scattering angle of 90°). The measurements were performed at room temperature with five runs for each determination. The zeta potential measurements were carried out at 25 °C on a Malvern Zetasizer 3000 HS instrument (Malvern Panalytical Ltd., Malvern, UK) after the same dilution.

#### 3.5.2. Entrapment Efficiency 

The Entrapment Efficiency (EE) of systems was determined by the dialysis method [49]. To evaluate the amount of bioactive ingredients encapsulated into the vesicles, samples (1 mL) were purified from the unincorporated components by dialysis (Spectra/Por^®^ membranes: 12–14 kDa MW cutoff) in water (0.5 L) for 2 h at room temperature, with distilled water refreshed after 30 min (2 L in total amount). At the end of the purification process, the antioxidant activity (Inhibition%) of the samples before and after dialysis was measured by DPPH assay and the EE was calculated as a percentage of the antioxidant activity after dialysis versus that before dialysis as reported in this formula: EE% = (Inhibition% dialyzed sample/ Inhibition% nondialyzed sample) × 100.

#### 3.5.3. Physical Stability

The physical stability of the lipid vesicles was assessed by monitoring the size and the PDI over 20 weeks of storage at +4.0 ± 1.0 °C in the dark to prevent extract oxidation and phospholipids hydrolysis [6]. For this study, at predetermined periods (0, 4, 8, 12, 16, and 20 weeks) aliquots of vesicle suspensions were diluted in ultrapure water (1:800; *v*/*v*) and the change in ethosome size and PDI were measured by DLS, as reported in the Section 3.5.1.

### 3.6. In Vitro Release Studies 

The polyphenol release profiles of lipid vesicles were carried out using a Franz-type static glass diffusion cell (15 mm jacketed cell with a flat-ground joint and clear glass with a 12 mL receptor volume; diffusion surface area = 1.77 cm^2^) equipped with a V6A Stirrer (PermeGearInc., Hellertown, PA, USA). A cellulose membrane (MF-Millipore cellulose nitrate 0.22 μm, Sartorius Stedim, Biotech GmbH, Germania) was placed between the receptor and the donor compartments and 12 mL of a mixture of 3:7 (*v/v*) ethanol/pH 7.4 buffer was used as the receptor medium. The donor compartment was filled with 1 mL of vesicle suspension. The systems were kept at 32.0 ± 1.0 °C under magnetic stirring (100 rpm/min). Aliquots (0.2 mL) were withdrawn at predetermined intervals, and the release medium was refilled with the same volume. The polyphenols from the released medium were determined by UV–vis spectrophotometry using the Folin–Ciocalteu method and compared with the free extract dissolution in the same medium. The polyphenol release profiles were performed in triplicate.

Kinetic analysis of the in vitro release data was estimated after fitting in different release models by using the corresponding equations, namely zero-order (cumulative percentage TPC release versus time), first-order (log cumulative percentage TPC remaining versus time), Higuchi’s (cumulative percentage TPC release versus square root of time), and Korsmeyer’s (log cumulative percentage TPC versus log time). The correlation coefficient (R^2^) was used as an indicator for the best fitting in the considered models.

### 3.7. Rosehip Polyphenols in Vitro Skin Retention Studies

In vitro skin retention studies were performed on Franz diffusion cells using porcine ear skin as a membrane model [51,52]. Porcine ears were provided by a local slaughterhouse (CLAI, Faenza (RA), Italy and full-thickness skin was isolated by using a method previously reported [53]. The excised circular pig skins with 1.60 mm thickness were placed between the donor and receptor compartments with the inner part of the skin facing the upper inside portion of the cell. Ethosomal suspension and free extract solution (1 mL) were loaded on the skin in the donor compartment. The receptor medium consisted of a 3:7 (*v*/*v*) ethanol/buffer pH 7.4 solution and it was maintained under magnetic stirring at a temperature of 32.0 ± 1.0 °C throughout the experiment. For the permeation studies aliquots (0.2 mL) were withdrawn after 1, 2, 4, 6, and 24 h from the receptor medium and immediately replaced with an equal volume of fresh medium. The Folin–Ciocalteu test was used to detect the presence of polyphenols on withdrawn aliquots. At the end of the 6 h or 24 h permeation study, the skin was removed from the Franz cells and the excess of formulation was gently removed using a cotton swab. Then, the skin was cut into tiny pieces, placed into separated beakers, and extracted with 5 mL of a mixture of ethanol/water 50/50 *v*/*v*. Beakers were covered to avoid the evaporation of the solvent and stirred at 300 rpm for 24 h at room temperature. The next day, the samples were centrifuged and spectrophotometrically analyzed by using the Folin–Ciocalteu method to determine the amount of TPC retained in the skin. The DPPH test was also performed to evaluate the antioxidant activity [52,53]. Six replicates were performed for each experiment. 

### 3.8. Cell Viability Studies

WS1 cells (American Type Culture Collection, ATCC, Manassas, VA, USA) were used and were grown routinely in 5% CO_2_/humidified air at 37 °C with Dulbecco’s modified Eagle medium supplemented with D-glucose (4.5 g/L), FBS (10% *v*/*v*), L-glutamine (2 mM), penicillin (1000 U/mL), and 1 mg/mL of streptomycin. Cells were seeded at 5.000 cells/well (15 × 10^3^ cell/cm^2^) in a 96-well plate (Corning^®^, Tewksbury, MA, USA) and incubated for 24 h to allow cell adhesion. After adhesion, the medium was removed and replaced with fresh medium containing the desired concentration of the compounds to be tested. 

To test the cell viability, MTT assay was performed according to Calonghi et al. [54]. Briefly after 24 h of treatment, 0.01 mL of MTT dissolved in DPBS at the concentration of 5 mg/mL was added to each well and incubated for 4 h at 37 °C in the dark. Then, the medium was removed, the reduced formazan crystals were dissolved in 0.1 mL pure isopropanol, and the absorbance at 570 nm was measured using a multiwell plate reader (Tecan, Männedorf, CH). Data were analyzed by Prism GraphPad v8 software (GraphPad Software, San Diego, CA, USA).

### 3.9. Statistical Analysis

All the experiments were performed in triplicate, except for in vitro retention studies, for which six replications were conducted. The results were expressed as mean ± standard deviation (S.D.). For all the performed studies, Student’s *t*-test was used to determine statistical significance. Differences were deemed significant for *p* < 0.05.

## 4. Conclusions

In recent years, demand for natural ingredients and sustainable cosmetic products has been constantly on the rise. For this reason, natural resources, including wild plants like *R. canina* L., which is rich in many bioactive compounds, play an important role in cosmetic industry. In this study, LC-DAD-MS/MS and ^1^H-NMR analysis of the RCL extract revealed the presence of catechins, cyanidin, quercetin, gallic acid, ascorbic acid, sugars, and other organic acids that can be considered useful cosmetic ingredients. However, to protect RCL extract from oxidation and to improve the skin retention of bioactive compounds, RCL extract was encapsulated in ethosomes that exhibited good physicochemical characteristics such as nanometric size, a narrow PDI, an EE% of 92.30 ± 0.02, and a good stability over 20 weeks. In addition, ethosomes showed a slow release of phytocompounds in comparison to the solution of rosehip extract and the percentage of extract retained in the skin after 24 h was 48.98 ± 1.77%. Moreover, the MMT assay showed that WS1 viability was not affected by the RCL extract free or RCL extract loaded ethosomes. In conclusion, our findings suggest that ethosomal formulations could be exploited as nanocarriers for the delivery of phenolic compounds of *R. canina* extract for cosmetic use. 

## Figures and Tables

**Table 1:** Content of ascorbic acid and phenolic compounds from the extract of RCF, RCD, and RCL.

Sample	Ascorbic Acid *	Catechin *	Cyanidin *	Quercetin *	Gallic Acid *
RCF	4.03 ± 0.32	7.79 ± 0.62	5.32 ± 0.49	3.01 ± 0.28	0.81 ± 0.18
RCD	4.48 ± 0.31	8.80 ± 0.59	5.91 ± 0.38	3.66 ± 0.24	0.88 ± 0.15
RCL	5.59 ± 0.36	12.27 ± 0.74	6.87 ± 0.42	4.38 ± 0.30	1.12 ± 0.13

* µg/mg extract ± SD.

**Table 2:** TPC, TFC, and AA% of *Rosa canina* L. extracts.

Type of Extract	TPC (μg GAE/mg Extract)	TFC (μg QE/mg Extract)	AA%
RCF	68.70 ± 0.46	23.16 ± 0.62	85.02 ± 0.47
RCD	88.71 ± 0.95	25.38 ± 0.49	74.50 ± 0.23
RCL	128.63 ± 1.03	26.43 ± 0.18	88.83 ± 0.70

**Table 3:** VS, PDI, ζ potential and EE of LP, HYA, and ET, unloaded and loaded with RCL extract.

	Size (nm)		PDI		ζ (mV)		EE%
	Unloaded	Loaded	Unloaded	Loaded	Unloaded	Loaded	Loaded
LP	375 ± 27	1971 ± 191	0.23 ± 0.19	0.45 ± 0.01	−66.66 ± 1.40	−47.22 ± 3.23	65.50 ± 0.29
HYA	451 ± 19	2236 ± 68	0.29 ± 0.10	0.32 ± 0.10	−83.59 ± 2.59	−56.17 ± 3.95	63.97 ± 0.35
ET	127 ± 1	196 ± 2	0.14 ± 0.40	0.20 ± 0.02	−46.99 ± 0.93	−37.36 ± 0.55	92.30 ± 0.02

**Table 4:** Release kinetic parameters for ET RCL.

Sample	Zero-Order (R^2^)	First-Order (R^2^)	Highuci (R^2^)	Korsmeyer–Peppas (R^2^)
ET RCL	0.977	0.969	0.951	0.976 (*n* ˃ 1.0)

## Data Availability

The data are contained within the article.

## Sample Availability

Samples of the compounds are not available from the authors.
